# The Role of Regional Anaesthesia and Acute Pain Services in Value-Based Healthcare

**DOI:** 10.4274/TJAR.2023.231478

**Published:** 2023-12-27

**Authors:** Sapna Ravindranath, Yatish S Ranganath, Kevin Backfish-White, John Wolfe, Sanjib Adhikary

**Affiliations:** 1 Indiana University Faculty of Medicine, Department of Anaesthesia, Indiana, USA; 2 Penn State College of Medicine, Department of Anaesthesiology and Perioperative Medicine, Pennsylvania, USA

**Keywords:** Acute pain service, operating room efficiency, opioid epidemic, perioperative outcomes, perioperative pain management, regional anaesthesia, value-based health care

## Abstract

Value-based healthcare prioritizes patient outcomes and quality relative to costs, shifting focus from service volume to delivered value. This review explores the significant role of regional anaesthesia (RA) and acute pain services (APS) within the evolving value-based healthcare (VBHC) framework. At the heart of VBHC is the goal to enhance patient outcomes while simultaneously optimizing operational efficiency and reducing costs. The review underscores the need for VBHC and illustrates how integrating RA/APS with Enhanced Recovery Protocols can lead to improved outcomes, aligning directly with the goals of the Triple Aim. Several clinical studies show that RA improves patient outcomes, enhances operating room efficiency, and reduces costs. This is complemented by a discussion on the integration of RA and APS into the VBHC model, highlighting emerging value-based payment structures and strategies for their successful implementation. By merging specialized RA/APS protocols with standardized clinical practices, significant improvements in operating room efficiency and associated economic benefits are observed. Across the healthcare spectrum, from providers to payers, this synergy results in enhanced operational efficiency and communication, raising the standard of patient care. Additionally, the potential of RA and APS to address the opioid crisis, through alternative pain management methods, is emphasized. Globally, the shift towards VBHC requires international collaboration, sharing of best practices, and efficient resource allocation, with RA and APS playing a crucial role. In conclusion, as healthcare moves toward a value-driven model, RA and APS become increasingly essential, signaling a future of refined, patient-centered care.

**Main Points**

• Regional anaesthesia (RA) and acute pain services (APS) are crucial in the value-based healthcare (VBHC) framework, significantly improving patient outcomes and operational efficiency.

• Studies demonstrate that RA enhances patient recovery and operational throughput, while also reducing healthcare expenses.

• Their integration into VBHC not only improves care quality but also provides alternative pain management strategies, crucial in addressing the opioid crisis.

• As healthcare pivots to value-driven models, RA and APS emerge as key strategies, influencing both patient care and healthcare economics. Research and its practical application in this area are vital for future advancements.

## Introduction

Value-based healthcare (VBHC) is a healthcare delivery model in which hospitals and physicians are compensated based on patient health outcomes, in contrast to the traditional fee-for-service model that pays for each service or procedure performed.^1,2^ The “value” in VBHC is derived from measuring health outcomes against the cost of delivering the services. VBHC prioritizes patient well-being, evidence-based practices, and cost efficiency, aligning with the Institute for Healthcare Improvement’s Triple Aim Framework which targets improved patient experience, enhanced population health, and reduced costs.^3^ As healthcare systems globally face challenges from scarce resources, escalating healthcare costs, and aging populations, there is a notable shift towards adopting VBHC to revolutionize health delivery and management. In the United States, the need for VBHC is highlighted by soaring healthcare costs, which reached $4.3 trillion in 2021, averaging $12,914 per person annually. Projections suggest this could nearly double by 2031, pushing healthcare’s GDP contribution from 18.3% in 2021 to 19.6%.^4^ Yet, despite such expenditures, the U.S. trails in life expectancy compared to several developed nations and faces an estimated 98,000 preventable deaths annually.^5^ This discrepancy between costs and outcomes further emphasizes the urgency for VBHC.

**Figure 1:**
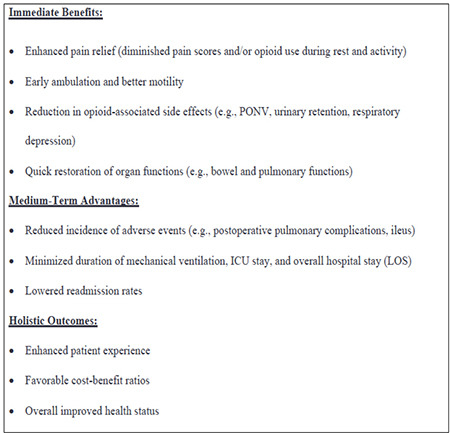
Enhancing healthcare value: a framework for RA/APS and ERP in the triple aim context. RA, regional anaesthesia; APS, acute pain service; ERP, enhanced recovery pathway; PONV, postoperative nausea and vomiting; LOS, length of stay.

Surgical care expenses represent a considerable segment of overall health care expenditure, with data from 2014 indicating that they accounted for as much as 51% of total Medicare spending.^6^ Surgery and anaesthesiology are inextricably linked, as most surgical interventions require anaesthesia. Anaesthesiologists, in collaboration with perioperative professionals, offer specialized skills ranging from preoperative assessments to postoperative surveillance in high-value surgical care. Their unique position in hospital-based care enables them to spearhead the recalibration of perioperative processes, improving operational efficiency and clinical outcomes. Such improvements benefit patients through reduced complications and costs, while also optimizing coordination, much to the advantage of hospital administrators and insurance payors. Regional anaesthesia (RA) is a subspecialty within anaesthesiology, utilizing neuraxial blocks (e.g., spinal, or epidural blocks) and peripheral nerve blocks for surgical procedures. RA is frequently chosen for its notable advantages, such as improved post-operative recovery, better postoperative analgesia, and reduced post-operative opioid use.^7^ Further, within the VBHC framework, RA in conjunction with Enhanced Recovery Pathways (ERPs) is gaining significance. Together, RA and ERPs aim to reduce complications and hospital stays and offer holistic improvements in patients’ experiences, perioperative expenses, and overall health status, aligning with the Triple Aim goals of VBHC (Figure 1).^8^

In this review, we will examine the role of RA and the Acute Pain Service (APS) in enhancing patient outcomes, bolstering healthcare efficiency, and cutting costs. Additionally, we will discuss their integration into the VBHC model and the benefits they present for various stakeholders. Finally, we will address future perspectives, emphasizing research opportunities and the implications for evidence-based practice.

### Role of RA and APS in Enhancing Patient Outcomes

Various RA techniques are available, including neuraxial blocks, paravertebral blocks, fascial plane blocks, and peripheral nerve blocks. A full exploration of every nerve block option for different surgical procedures is beyond the scope of this article. Nonetheless, when viewing anaesthesia care through the lens of VBHC, it becomes essential to underline the critical role, impact, and benefits of RA &amp; APS. This article will highlight these aspects, drawing from pertinent literature and using tables to summarize the main points (Tables 1 and 2).

### Summary of Studies on Patient Outcome Benefits (Table 1)

In thoracic surgeries, RA improves pulmonary function after lobectomy and reduces the risk of post-operative pulmonary complications and mortality, especially in chronic obstructive pulmonary disease patients.^9,10^ RA reduces unplanned intensive care unit (ICU) admissions, the duration of mechanical ventilation, and the length of ICU stays.^11^ Some studies also pointed to a potential reduction in the incidence of post thoracotomy pain syndrome.^12^ In cardiac surgeries, opioids traditionally took precedence over epidurals and blocks, mainly because of anticoagulation and hemodynamic concerns. However, the emergence of fascial plane blocks is shifting this trend. Blocks targeting the erector spinae and parasternal regions have been associated with improved recovery, greater patient satisfaction, and reduced ICU length of stay, though further research is warranted.^13,14^ Within vascular surgery, RA is associated with improved vessel patency, reduced failures, and eased transitions from arteriovenous graft to arteriovenous fistula post-dilation, attributed mainly to the sympathectomy effects of nerve blockade.^15^ For carotid endarterectomy, RA was deemed more cost-effective than general anaesthesia (GA) and was accompanied by shorter operative durations, reduced hospital stays, and fewer complications.^16^ Recent research also credited RA with lower in-hospital mortality rates in comparison to GA.^17^ Overall, for high-risk vascular surgery patients, RA often outperformed GA, though GA continues to be a dependable option when RA is not feasible.

RA is extensively used for orthopedic surgeries. In joint replacement surgeries, RA is associated with enhanced immediate post-operative pain relief, reduced blood loss, and fewer transfusions.^18^ Furthermore, a decreased risk of both major and minor complications was observed with spinal anaesthesia.^19^ Neuraxial anaesthesia, often combined with peripheral nerve blocks, improves readiness for discharge by reducing pain, opioid use, and post-operative nausea and vomiting, making it a frequent choice for same-day discharge arthroplasties.^20^ Peripheral nerve block catheters, used either in inpatient or ambulatory settings, have been shown to provide superior analgesia for up to 48 hours after orthopedic surgery.^21^ This analgesic benefit facilitates early initiation of rehabilitation and physiotherapy, potentially facilitating some surgeries to transition to a day-care model, offering cost efficiencies.^21,22^ Broadly, in orthopedic surgeries, peripheral nerve blocks (PNBs) enhance post-operative pain control, reduce opioid use and associated side-effects, shorten hospital stays, allow earlier initiation of physical therapy, cut readmission rates, improve patient satisfaction, and prevent unplanned pain admissions.^23^

In major abdominal surgeries-covering gastrointestinal, hepatobiliary, and urological procedures-varied RA techniques, such as thoracic epidural anaesthesia (TEA), intrathecal morphine, and fascial plane blocks like transversus abdominus plane and quadratus lumborum blocks are employed.  Some of these techniques reduce respiratory complications and resting pain scores.^24,25^ Moreover, they facilitate a quicker return of bowel function, though their impact on hospital length of stay remains debated.^26^ In addition, for open abdominal aortic aneurysm (AAA) surgeries, RA led to diminished blood loss and faster post-operative mobilization.^24^ In gastrectomies and esophagectomies, RA was associated with decreased dynamic pain scores and the previously mentioned benefits.^27^

In trauma patients, for managing rib fractures, techniques like TEA, paravertebral catheters, serratus anterior plane, and erector spinae block catheters reduced pain, with some also decreasing opioid use and delirium risk.^28,29^ While TEA might shorten mechanical ventilation, its broader efficacy remains debated.^30^ For hip fractures, nerve blocks effectively alleviate pain. Additional benefits include reduced pneumonia risk and quicker mobilization.^31^ In elderly hip fracture patients undergoing surgery, both RA and GA showed comparable post-operative results, emphasizing individualized choices.^32^

Beyond RA techniques, ketamine infusions overseen by APS can benefit patients experiencing significant post-operative pain, those tolerant to opioids, and individuals with conditions such as sickle cell disease or obstructive sleep apnea.^33^ Additionally, transitional pain clinics, often viewed as extensions of the APS, tackle postoperative and procedural pain, forming a bridge between hospitals and the community.^34^ These clinics, developed in response to escalating costs of chronic pain management and the opioid crisis, emphasize non-opioid approaches. Anaesthesiologists and APS specialists handle complex surgical patients at risk of persistent postoperative pain, including those on medications such as buprenorphine or methadone, during the perioperative phase.

### Role of RA & APS in Enhancing Health Care Efficiency and Decreasing Costs

RA techniques not only enhance patient outcomes-by reducing pulmonary complications, shortening ICU stays, improving AV fistula survival, and promoting early bowel recovery but also drive cost-effectiveness and heightened efficiency. In this section, we will further explore these fiscal and operational advantages, highlighting a few studies that underscore the economic and efficiency benefits of RA (Table 2).

### Summary of Studies on Efficiency and Cost-Effectiveness

A systematic review of 28 studies involving 27,581 patients found that RA in ambulatory surgery resulted in lower overall hospital costs. This decrease was largely due to reduced OR times, faster post-anaesthesia recovery, and shorter hospital stays.^35^ Another review, involving 8,888 patients, reported that among 3,364 patients who used parallel processing with RA, there was a reduction of anaesthesia-controlled time (ACT) by 10.4 minutes and turnover time by 16.1 minutes. Furthermore, Postanesthesia Care Unit (PACU) time was shortened by 26.6 minutes, allowing for an increase in daily OR throughput by 1.7 cases on average.^36^

Multiple studies have demonstrated that the optimization of systems to support RA and APS services can improve OR efficiency and throughput. In a study of 993 joint arthroplasty patients, introducing a RA block room reduced OR time by 23 minutes and ACT by 20 minutes; the use of peripheral nerve blocks increased from 63.1% to 87.0%; 1 additional surgery was added each day.^37^ In another study comparing 688 traditional cases to 905 high-throughput cases in joint arthroplasties, the introduction of an adjacent “induction room” and other systematic changes increased the number of surgeries from 2.6 to 3.4 per room per day. Additionally, nonoperative time decreased by 36 minutes, operative time by 14 minutes, and contribution margins increased by 19.6%.^38^ In a cohort of 328 hand and wrist surgeries using two operating rooms, reductions of 23 and 20 minutes were observed in OR and ACT times, respectively.^39^ A 12-month retrospective study of 254 patients revealed that using thoracic epidural placements in a preoperative block room led to a net OR time saving of 19.1 minutes and reduced the epidural failure rate from 16.0% to 5.6%.^40^

Examining studies that focus on cost-effectiveness, an analysis of 14,713 anterior cruciate ligament (ACL) repair patients reported an average surgical cost of $24,707, with increases associated with the use of GA alone without RA, Hispanic ethnicity, and the presence of multiple chronic conditions.^41^ In another study, among 154 ACL reconstruction patients, those with RA had fewer unexpected hospital admissions, shorter PACU stays, reduced opioid consumption, and quicker discharge readiness.^42^ In a study of 346 carotid endarterectomy patients, RA proved to be more cost-effective, with costs amounting to $7,122 as opposed to $10,140 for GA. Additionally, RA was associated with shorter operative and hospital times.^43^ A 2016 study of 120 patients compared the costs of continuous popliteal sciatic nerve block for foot surgery and found cost benefits in outpatient surgery settings without compromising patient outcomes.^44^

### Integration of RA and APS with VBHC

Integrating RA and APS into VBHC focuses on improving patient outcomes and optimizing resources. VBHC rewards quality over volume, ensuring transparent outcome reporting and patient-centered care. Understanding this integration demands insight into the healthcare delivery transition. The shift from traditional fee-for-service to value-based payment models has given rise to several VBHC models. Bundled Payments offer a fixed price for a service bundle, while Shared Savings Programs, like the Medicare Shared Savings Program, incentivize cost-saving. Capitation involves a fixed monthly payment to physicians for specified services. Other notable models include Pay for Performance, such as CMS’s Hospital Value-Based Purchasing Program, and Patient-Centered Medical Homes that prioritize care coordination; Value-Based Contracting linking drug payments to effectiveness; the Global Budgets model, such as Maryland’s All-Payer Model; and Shared Risk Models involving both savings and losses.

In surgery, bundled payments are prominent. They consider expenses from preoperative to postoperative care, exemplified by CMS’s Bundled Payments for Care Improvement and Comprehensive Care for Joint Replacement models. However, its adoption varies by region, procedure, and healthcare setting. While fee-for-service persists, there’s a trend towards value-based systems, sometimes blending in hybrid models.

For successful RA/APS and VBHC integration, identifying optimal patient groups and crafting tailored RA and APS protocols is paramount. Collaborative teams of surgeons, anaesthesiologists, pain experts, and other professionals ensure thorough pain management. Establishing metrics for RA and APS efficiency, centered on patient recovery and satisfaction, remains essential.

### Benefits of VBHC for the Stake Holders

RA/APS offer substantial advantages to the core participants in today’s healthcare ecosystem, which includes patients, healthcare providers, healthcare system administration (HSA), payers, employers, and vendors. For patients, VBHC provides a quicker recovery process paired with cost-efficiency. The focus on prevention results in fewer doctor visits, fewer medical procedures, and less medication cost. Integrating RA/APS into this model provides patients with advanced pain management strategies such as nerve blocks, offering superior benefits over opioids, as extensively discussed in the preceding sections. Moreover, RA/APS providers work to comprehend and address patient expectations, simplifying medical jargon and enhancing communication and trust.

Healthcare providers, including RA/APS professionals, experience enhanced operational efficiency as VBHC emphasizes streamlined, consistent protocols. The synergy between RA/APS, surgical, and nursing teams augments operating room productivity, conserving both time and money. Additionally, the shift from volume to value propels providers to champion quality, assuring superior clinical results. HSAs greatly benefit from integrating RA/APS into a VBHC framework. Adopting specialized protocols not only enhances operating room efficacy by reducing ACT and turnover time but also optimizes metrics like PACU, refining patient flow, as supported with evidence in previous sections. This reduces staff overtime and consequently lowers operational expenses. Elevating the contribution margin via increased daily case numbers during regular operational hours bolsters the system’s financial health. Notably, RA/APS’s role in curbing hospital readmissions due to pain issues can boost a facility’s value base purchasing status.

For payers within VBHC, there’s an opportunity to achieve superior cost management through bundled payments, while also reducing risks. RA/APS services, through strategic alliances, present enhanced communication, and data sharing, offering a holistic view of patient care and potential financial gains. The rise of value-centered initiatives places RA/APS firmly within the broader perioperative context, turning hurdles into opportunities. By cultivating strategic partnerships in frameworks like the perioperative surgical home (PSH) and enhanced recovery after surgery, and establishing strong localized data management systems, RA/APS emerges as a pivotal entity during detailed contract talks. Employers, vital in healthcare due to their role in insurance provision, recognize the value in the cost reductions RA/APS’s efficient care pathways bring. These pathways speed up an employee’s return to work and minimize disability durations, resulting in enduring fiscal advantages. Lastly, vendors, particularly pharmaceutical companies, and medical device manufacturers, can align their product prices with the real value they deliver to patients within VBHC. The emphasis on actual outcomes enables vendors to market their products more efficiently.

### Future Horizons: Role of RA and APS in Advancing VBHC

The last two decades have witnessed a paradigm shift in healthcare, evolving from a volume-driven approach to one rooted in value. The essence of VBHC is to deliver optimal health outcomes for every dollar spent, placing the patient squarely at the center of this framework. As Porter and Teisberg suggested in 2006, the aim is to align healthcare providers and payers with the objective of enhancing patient outcomes while managing costs.^2^ However, this transition, though gaining momentum, faces several challenges: a value crisis where costs rise without corresponding improvement in outcomes;^45^ an evidence crisis, characterized by the rapid expansion of biomedical knowledge but slow integration into clinical practice;^46^ and a purpose crisis, seen in the widening gap between healthcare professionals’ ideals and their working realities.^47^

In this evolving landscape, RA and APS play a pivotal role. Over 100 million Americans grapple with chronic pain, leading to treatments costing over $635 billion annually surpassing expenses for heart disease and cancer.^48^ The opioid crisis, partly stemming from postoperative opioid prescriptions, highlights the pressing need for alternative pain management strategies. The transformative potential of RA is evident here. As anaesthesiologists, integrating RA into perioperative pain medicine can significantly reduce opioid prescriptions, especially when collaborating with comprehensive surgical teams. Institutions combining anaesthesia pain management services with multimodal analgesia and RA observe notably improved postoperative pain control and reduced opioid dependency. Moreover, RA’s advanced techniques can streamline patient discharge plans and offer evidence-based guidelines for opioid prescriptions when necessary. This approach not only improves patient outcomes but also boosts the overall value in healthcare delivery. The Michigan Opioid Prescribing Engagement Network-OPEN initiative fosters evidence-based practices to minimize perioperative opioid use by uniting hospitals and payers. This partnership standardizes protocols, curtails excessive opioid prescriptions post-surgery, and ensures safer pain management for patients statewide.

The integration of RA and APS into VBHC extends beyond immediate opioid reduction. It signifies a move towards future-oriented medicine that prioritizes research and evidence-based practice. Highlighting a holistic approach, the PSH provides a comprehensive view of surgical care, with pain management as a primary focus.^49^ Transitional pain clinics serve as a bridge between immediate postoperative pain relief and long-term pain prevention strategies.^34^ Collectively, these models advocate a coordinated approach that elevates patient outcomes, focusing on pain management. Such endeavors emphasize the need for ongoing research, especially in customizing RA techniques to individual patient needs, adjusting interventions for specific surgical procedures, and understanding the long-term benefits of RA in averting chronic pain and reducing hospital readmissions. Within VBHC’s broader framework, in-depth research into RA promises to decrease complications, enhance patient satisfaction, and judiciously allocate resources.

Governments and healthcare institutions worldwide are acknowledging the need to integrate VBHC objectives with clinical practice. As observed in the Netherlands and Singapore, strategies prioritize outcomes-based healthcare, emphasizing the importance of standardized outcome metrics and value-based payment models.^50^ However, fully realizing VBHC’s potential depends on global collaboration, exchanging best practices, and consolidating resources. Initiatives like the Global Coalition for Value in Healthcare under the World Economic Forum illustrate the promising future that awaits.

In conclusion, as healthcare transitions to value-centric models, RA and APS emerge as foundational strategies that profoundly impact patient outcomes and the financial landscape of healthcare, rather than mere adjuncts. Their integration into the VBHC framework not only elevates the quality of care, reduces costs, and enhances efficiency but also offers a guiding light in addressing some of the most pressing challenges in modern medicine. Emphasizing research in this area and translating findings into clinical practice will be pivotal in shaping the future of healthcare.

## Figures and Tables

**Table 1:**
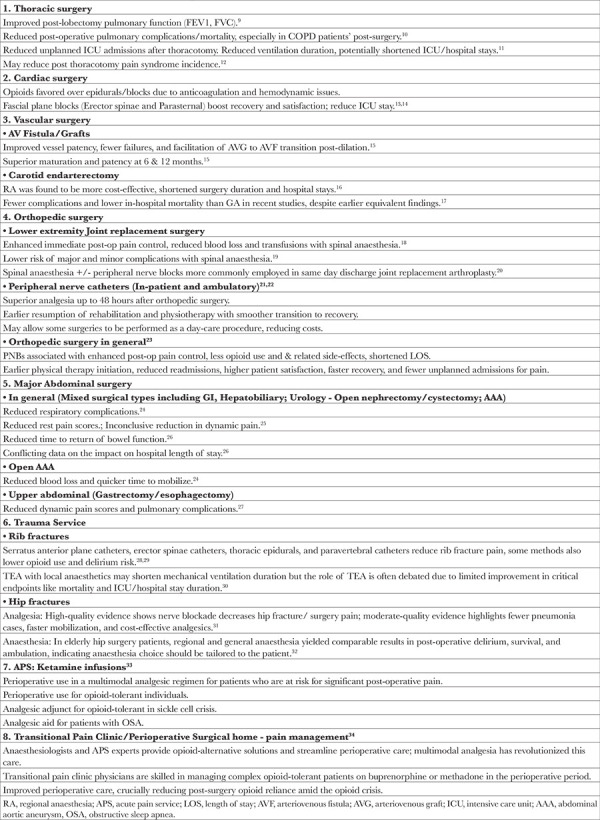
RA/APS Studies: Evidence for Improved Patient Outcomes

**Table 2:**
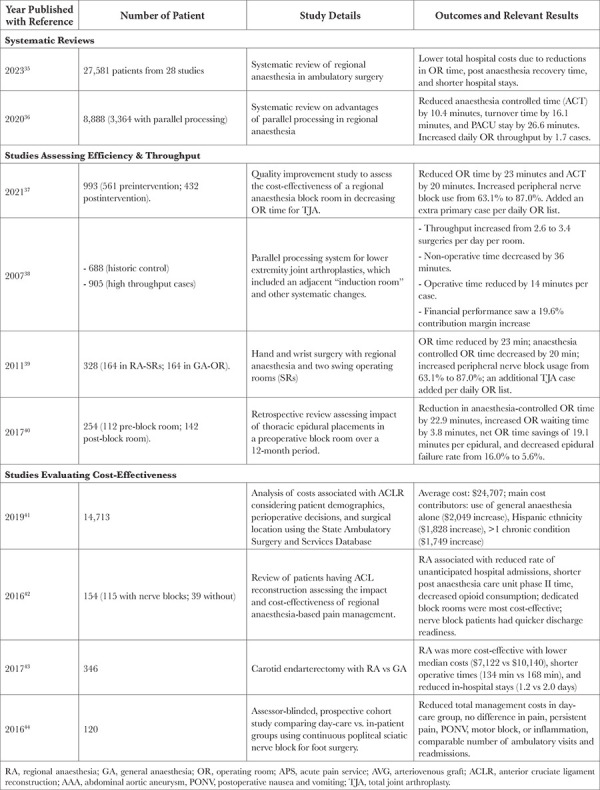
RA & APS Studies: Evidence for Enhancing Efficiency and Decreasing Costs
